# Germination Stage Oxygen Deficiency (GSOD): An Emerging Stress in the Era of Changing Trends in Climate and Rice Cultivation Practice

**DOI:** 10.3389/fpls.2016.00671

**Published:** 2016-05-18

**Authors:** Soham Ray, Joshitha Vijayan, Ramani K. Sarkar

**Affiliations:** ^1^National Rice Research InstituteCuttack, India; ^2^Integrated Rural Development and Management, Ramakrishna Mission Vivekananda UniversityKolkata, India

**Keywords:** rice, direct seeding, submergence, Germination Stage Oxygen Deficiency (GSOD), anaerobic germination, global climate change

## Introduction

Rice (*Oryza sativa*) is probably the most important crop to ensure world's food security. Adapted to semi-aquatic ecology, it has got an innate ability to tolerate submergence to some extent. Nevertheless, long period of submergence invites unavoidable negative consequences starting from yield and quality reduction of the produce to complete crop failure. Under submergence plants are deprived of oxygen either completely (anoxia) or partially (hypoxia) which is well-recognized as a potent abiotic stress factor. Significant research efforts have been directed globally toward understanding the mechanism of submergence tolerance particularly during vegetative stage of rice; as the crop frequently encounters flash flood or water stagnation after transplanting and during its initial growth stage. Thanks to these scientific endeavors which resulted in identification, cloning, characterization, and deployment of genes like *SUB1* (Xu et al., 2006; imparts quiescence during submergence) and *SNORKEL1* and *SNORKEL2* (Hattori et al., 2009; induce quick elongation of internodes) which help rice plants either to tolerate or to escape submergence during its vegetative growth stage. However these genes, unfortunately, have little or no consequences when seed germination under submergence is concerned.

Seed germination under oxygen deprived condition is gradually becoming an area of active research owing to the shifting trend of direct seeding by abandoning traditional transplanting method, in order to intensify as well as economize rice cultivation (Kumar and Ladha, 2011). Though, direct seeding can potentially reduce cultivation cost, it also makes the crop vulnerable to the fluctuations of monsoon rains which are quite frequent in South and South-East Asia (the rice bowl of the world) dominated by low land rain-fed ecosystem. Unpredicted heavy downpour immediately after direct seeding can call upon flash flood. Low land situation can also result in continuous water stagnation for several days. Stagnant water can condition typical stress situation by restricting free diffusion of oxygen from air to germinating seeds (Narsai et al., 2015). But grossly being adapted to aquatic ecology, rice has developed the unique mechanism to germinate and elongate its coleoptile under water (nearly at the rate of 1 mm h^−1^) even in complete absence of oxygen (Magneschi and Perata, 2009; Narsai et al., 2015)—a phenomenon termed as anaerobic germination (AG). However, anaerobic germination potential (AGP) varies greatly among different rice cultivars which ultimately provide an edge to a few cultivars to perform better under oxygen deprived conditions over others. In recent literatures, the rice cultivars having better germination potential under oxygen deprived condition and hence are capable of withstanding the stress have been deemed as anaerobic germination tolerant, while the cultivars having the contrasting character have been termed as anaerobic germination susceptible (Angaji et al., 2010; Baltazar et al., 2014; Kretzschmar et al., 2015).

Since, this is an upcoming area of research, it is necessary to set the terminologies right at the very beginning as once established these terminologies will stand for future and can even percolate and perpetuate in related field of studies. In this article, we would try to critically analyze the aptness of the terminologies used so far by logical arguments and present our view where we feel a need for change/rectification.

## Anaerobic respiration: The key to germinate under oxygen deficiency

Seed germination is a highly energy demanding process and the required energy is obtained by catabolizing the reserved food contained in the seed itself. In normoxic condition (normal oxygen concentration), where there is no hindrance in free diffusion of oxygen from air to the germinating seed, aerobic respiration of the stored food reserve is the chief mode of deriving the required energy. But as we have mentioned before, submergence hinders free oxygen diffusion, and abiotic stress gets induced in the process, technically termed as anoxia (no oxygen) or hypoxia (3% oxygen), by restricting free diffusion of oxygen from air to germinating seeds (Narsai et al., 2015). Anoxia is an extremely rare situation which hardly occurs during flash flood or even during long period of water stagnation in agricultural fields, whereas hypoxia is more a real-life problem during submergence. Nevertheless, during such oxygen deficient condition (in whatever form it might be), the energy required for germination is obtained from the alternate resort i.e., by anaerobic respiration. Hence, “ANAEROBIC GERMINATION (AG)” is an apt terminology in our opinion which signifies the inherent capacity of seed to GERMINATE under oxygen deficiency by obtaining the required energy through ANAEROBIC respiration. It is also justified to use the terminology “ANAEROBIC GERMINATION POTENTIAL (AGP)” by drawing an analogy with the basic concept of “GERMINATION POTENTIAL” frequently used in the field of seed science.

## Anaerobic germination: A stress or a way to escape stress?

**Figure 1:**
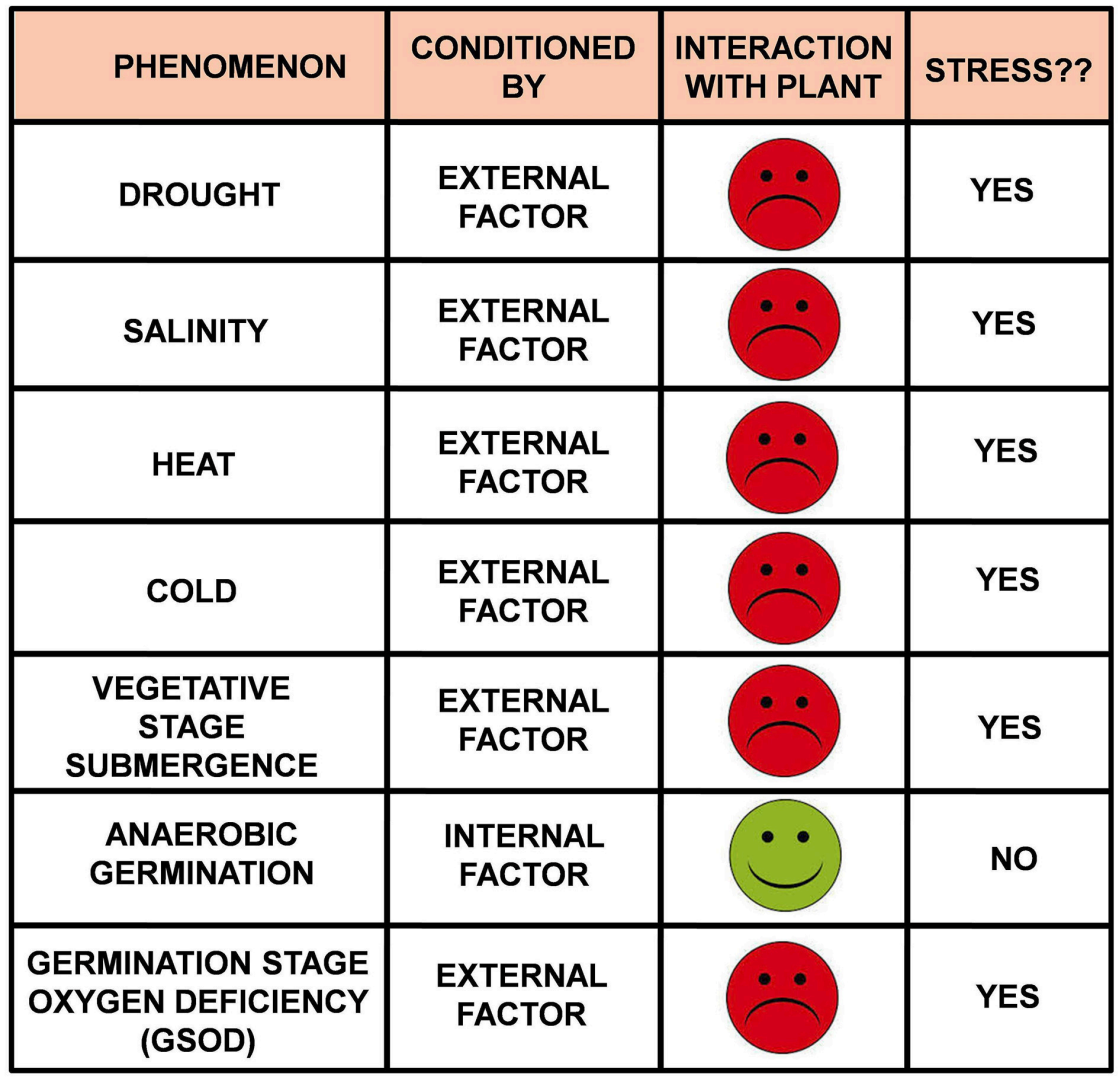
**“Under stress” or “not under stress” that is the question!** If a plant (or any organism) showing any abnormalities is under stress or not can be judged by answering two simple questions: **Question 1:** what conditions the abnormality? **Probable answers:** (A) Internal factor(s) or (B) External factor(s); **Question 2:** What is the physiological interaction of the plant with the causal factor(s)? **Probable answers:** (A) Positive (green smiley) or (B) Negative (Red frowney); If **Answer B** fits both the questions then the plant (or the organism) is **under stress**.

In some of the recent literatures the term “ANAEROBIC GERMINATION TOLERANT” has been designated to the seeds having good AGP, hence can germinate and survive under submerged conditions (Angaji et al., 2010; Baltazar et al., 2014; Kretzschmar et al., 2015). Consequently the seeds have relatively poor AGP and succumb under similar condition have been regarded as “ANAEROBIC GERMINATION SUSCEPTIBLE.” We discern that, these are not the proper terminology to use. “Tolerance” and “susceptibility” are the terms associated with “stresses”—the one which endures and survives it is regarded as “tolerant” while the one which succumbs to it is regarded as “susceptible.” These two words (“tolerant” and “susceptible”) are generally preceded by the stress under question. For example, in case of drought stress, “drought tolerant” is the one which can successfully overcome drought situation while those which get perished under drought are termed as “drought susceptible.” Similarly comes “cold tolerant and cold susceptible,” “heat tolerant and heat susceptible,” “salinity tolerant and salinity susceptible” and so on. If we follow the same notion then “anaerobic germination” becomes a stress. Can “anaerobic germination” be regarded as a stress? “Stress” in plant physiology is defined as “any EXTERNAL FACTOR that NEGATIVELY influences plant growth, productivity, reproductive capacity, or survival” (Rhodes and Nadolska-Orczyk, 2001). “Anaerobic germination” is neither an external factor nor it influences negatively the basic physiology of the plant in any ways. Rather, it is purely an internal factor which helps the seeds to germinate under oxygen deprivation caused by submergence and hence must be considered as a positive influence beyond any doubt. So, the logic says “anaerobic germination” certainly is not a stress (Figure 1) rather an alternate mechanism to escape stress.

## Germination stage oxygen deficiency (GSOD): A stress newly defined

In that case, the next big question is what would be the appropriate terminology to regard this stress? Can this be regarded as “submergence”? In our opinion it won't be apt to do so. Basically, we see two reasons for that. Firstly, submergence for a brief period might not induce stress during germination unless there is a deficiency of oxygen. Such an oxygen deficiency occurs only when the seed is buried deep in the submerged soil (in reduced zone). Classic experiments have also demonstrated improved germination of seeds in such cases when they are coated with calcium peroxide (CaO_2_), a chemical compound which slowly releases oxygen in presence of water (Ota, 1982; Biswas et al., 2001). Similar oxygen deficiency can be created artificially in a nitrogen atmosphere (atmosphere saturated with nitrogen and hence lacks oxygen) where seed germination takes place anaerobically too (Kennedy et al., 1980). These tell quite clearly that not the submergence but the oxygen deficiency is the actual stress during germination. Secondly, “submergence” is well-known as stress during vegetative growth phase but the response of the plant to submergence during that stage is quite different from that of the germinating seed under submergence. So, in order to avoid confusions (at least for the beginners!) between these two different situations it is better to coin a new terminology to define the submergence stress during germination. Some authors have used the term “tolerance to anaerobic condition” (Miro and Ismail, 2013; Septiningsih et al., 2013). Anaerobic condition can be considered as stress as the natural process of germination requires free oxygen (aerobic process) and absence of which affect the normal process of seed germination negatively. Needless to mention, it is an external factor too. But still the term “tolerance to anaerobic condition” does not specifically state the fact that the anaerobic condition is created during germination. Since we have already argued that oxygen deficiency during germination should be dealt separately, we propose to use the terminology “Germination Stage Oxygen Deficiency” which can be abbreviated as “GSOD” to define this stress under question as it restricts itself strictly to the germination stage thus avoiding any confusion and also encompasses both anoxia and hypoxia. Let us justify this newly coined terminology in term of the definition of stress. “GSOD” actually indicates to the oxygen deficiency in the microenvironment of the germinating seed conditioned by submergence (or created artificially by saturating the environment by nitrogen). So, it is purely an external factor. And since it hinders normal process of seed germination, it affects the physiology of the plant negatively. So, it perfectly fits to the definition of stress (Figure 1). The seeds having high AGP will prevail under GSOD hence can be deemed as “GSOD tolerant;” while the seeds having low AGP will succumbs under GSOD hence can be regarded as “GSOD susceptible” (Figure S1). The quantitative trait loci (QTLs) conditioning GSOD tolerance have been regarded as *qAG*; for example, *qAG7* (Baltazar et al., 2014), *qAG2.1* (Baltazar et al., 2014), *qAG-9-2* (Angaji et al., 2010), *qAG7.1* (Septiningsih et al., 2013), etc. These QTLs impart GSOD tolerance to the cultivar containing them and the tolerance can be transferred by transferring these QTLs from GSOD tolerant to GSOD susceptible backgrounds. We feel that “*qAG*” may be continued as the signature for QTLs that indicates the innate ability of the seeds to germinate under anaerobic condition and hence is directly related to AGP.

## Concluding remarks

Oxygen deficiency can be fatal during germination. GSOD, as a stress, is gaining importance in rice cultivation due to shifting trend toward direct seeding. Its importance seems to be paramount when the fluctuation in monsoon rains due to global environment change is considered. Stress induced by oxygen deficiency during germination is an emerging field of study where very little have been discovered so far and hence a lot is expected to be revealed in near future. As a new field of study grows it coins several new terminologies to express or define the advancements or issues strictly related to that field. As we have mentioned at the very beginning, this article was intended to critically analyze the terminologies which are being used at present in this emerging field of study. We have made our best efforts to scientifically analyze all the frequently used terminologies. Some of the terminologies, we felt, are apt whereas few others seemed to be incorrect. We put forward new terminologies for that and tried to support them with logical scientific argument. We hope that these will be well-accepted by the global scientific community working in this field.

## Author contributions

SR conceived the idea and prepared the Manuscript. JV gave scientific input while preparing the draft. RS is working on the physiology of anaerobic germination of rice for quite some time and shared his experience in terms of physiological perspective while writing the manuscript.

## Funding

National Initiative on Climate Resilient Agriculture (EAP-158), Indian Council of Agricultural Research, New Delhi.

### Conflict of interest statement

The authors declare that the research was conducted in the absence of any commercial or financial relationships that could be construed as a potential conflict of interest.

## References

[B1] Angaji S. A. Septiningsih E. M. Mackill D. J. Ismail A. M. (2010). QTLs associated with tolerance of flooding during germination in rice (*Oryza sativa* L.). Euphytica 172, 159–168. 10.1007/s10681-009-0014-5

[B2] Baltazar M. D. Ignacio J. C. I. Thomson M. J. Ismail A. M. Mendioro M. S. Septiningsih E. M. (2014). QTL mapping for tolerance of anaerobic germination from IR64 and the aus landrace Nanhi using SNP genotyping. Euphytica 197, 251–260. 10.1007/s10681-014-1064-x PMC6711729 31481831

[B3] Biswas J. K. Ando H. Kakuda K. Purwanto B. H. (2001). Effect of calcium peroxide coating, soil source, and genotype on rice (*Oryza sativa* L.) seedling establishment under hypoxic conditions. Soil Sci. Plant Nutr. 47, 477–488. 10.1080/00380768.2001.10408412

[B4] Hattori Y. Nagai K. Furukawa S. Song X. L. Kawano R. Sakakibara H. (2009). The ethylene response factors SNORKEL1 and SNORKEL2 allow rice to adapt to deep water. Nature 460, 1026–1030. 10.1038/nature08258 19693083

[B5] Kennedy R. A. Barrett S. C. Vander Zee D. Rumpho M. (1980). Germination and seedling growth under anaerobic conditions in *Echinochloa crus-galli* (barnyard grass). Plant Cell Environ. 3, 243–248.

[B6] Kretzschmar T. Pelayo M. A. F. Trijatmiko K. R. Gabunada L. F. M. Alam R. Jimenez R. (2015). A trehalose-6-phosphate phosphatase enhances anaerobic germination tolerance in rice. Nat. Plants 24, 15124 10.3410/f.725735765.793509252 27250677

[B7] Kumar V. Ladha J. K. (2011). Advances, in Agronomy, Vol. 111, ed Sparks D. L. (Cambridge, MA: Elsevier Academic Press Inc), 297–413.

[B8] Magneschi L. Perata P. (2009). Rice germination and seedling growth in the absence of oxygen. Ann. Bot. 103, 181–196. 10.1093/aob/mcn121 18660495 PMC2707302

[B9] Miro B. Ismail A. M. (2013). Tolerance of anaerobic conditions caused by flooding during germination and early growth in rice (*Oryza sativa* L.). Front. Plant Sci. 4:269. 10.3389/fpls.2013.00269 23888162 PMC3719019

[B10] Narsai R. Edwards J. M. Roberts T. H. Whelan J. Joss G. H. Atwell B. J. (2015). Mechanisms of growth and patterns of gene expression in oxygen-deprived rice coleoptiles. Plant J. 82, 25–40. 10.1111/tpj.12786 25650041

[B11] Ota Y. (1982). Promotion of emergence and establishment of rice seedlings by using calcium peroxide-coated seeds in direct sowing on flooded paddy fields. Jap. Agric. Res. Q. 15, 221–226.

[B12] Rhodes D. Nadolska-Orczyk A. (2001). Plant stress physiology, in Encyclopaedia of Life Sciences (Nature Publishing Group). Available online at: http://www.els.net

[B13] Septiningsih E. M. Ignacio J. C. I. Sendon P. M. D. Sanchez D. L. Ismail A. M. Mackill D. J. (2013). QTL mapping and confirmation for tolerance of anaerobic conditions during germination derived from the rice landrace Ma-Zhan Red. Theor. Appl. Genet. 126, 1357–1366. 10.1007/s00122-013-2057-1 23417074

[B14] Xu K. Xu X. Fukao T. Canlas P. Maghirang-Rodriguez R. Heuer S.. (2006). Sub1A is an ethylene-response-factor-like gene that confers submergence tolerance to rice. Nature 442, 705–708. 10.1038/nature04920 16900200

