# Supplementary figures and images for: Germination Stage Oxygen Deficiency (GSOD): An Emerging Stress in the Era of Changing Trends in Climate and Rice Cultivation Practice
Source: Front Plant Sci. 2016 May 18;7:671. doi: 10.3389/fpls.2016.00671 (PMC4870248; doi:10.3389/fpls.2016.00671)

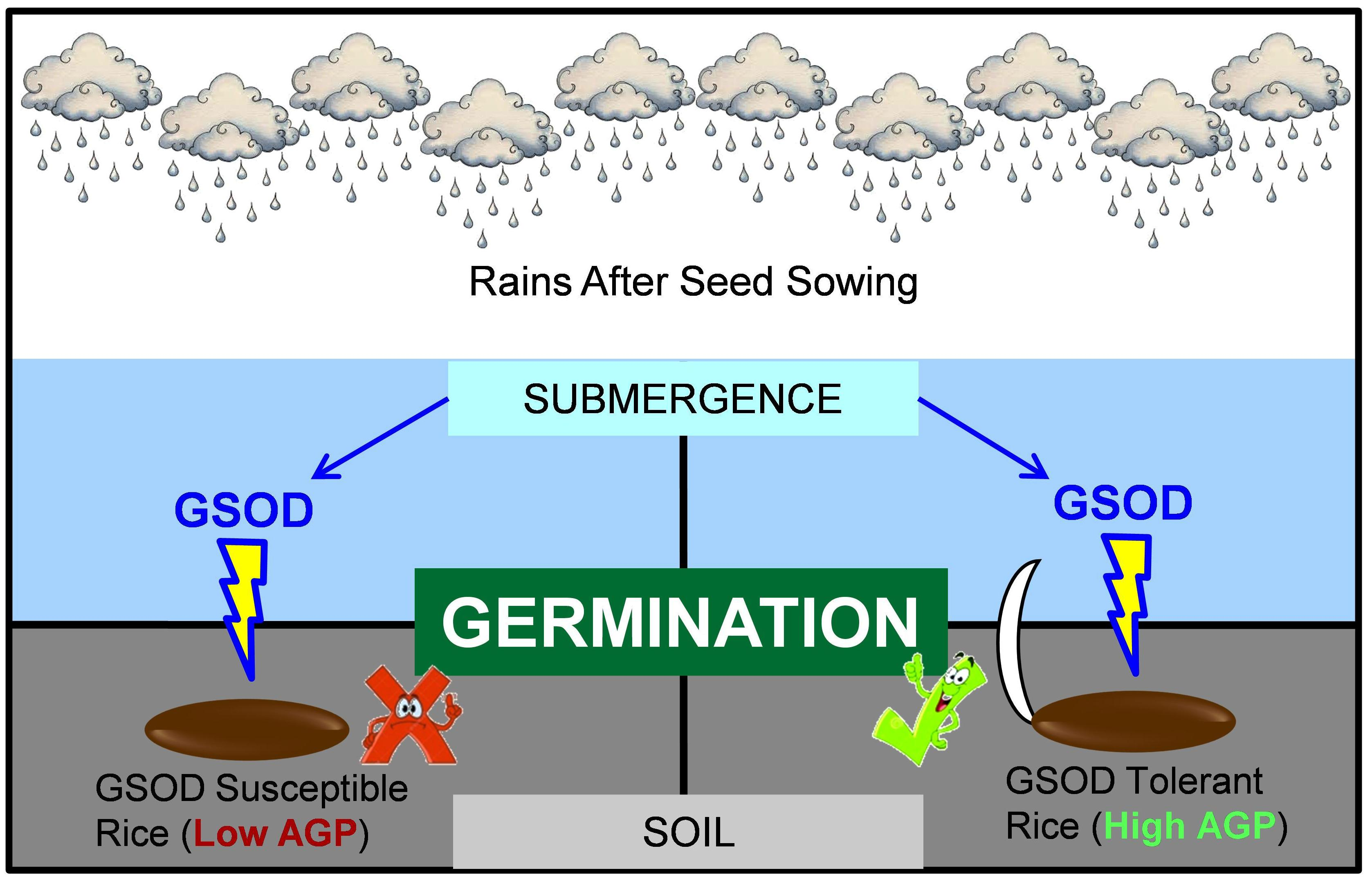

Supplement: Supplementary file 1 [file Image1.TIF]
